# The Association of Domestic Incense Burning with Hypertension and Blood Pressure in Guangdong, China

**DOI:** 10.3390/ijerph14070788

**Published:** 2017-07-14

**Authors:** Xiuling Song, Wenjun Ma, Xiaojun Xu, Tao Liu, Jianpeng Xiao, Weilin Zeng, Xing Li, Zhengmin Qian, Yanjun Xu, Hualiang Lin

**Affiliations:** 1 Guangdong Provincial Center for Disease Control and Prevention, Guangzhou 511430, China; songxl918@163.com (X.S.); xu-yd@163.com (X.X.); 2 Guangdong Provincial Institute of Public Health, Guangdong Provincial Center for Disease Control and Prevention, Guangzhou 511430, China; mawj@gdiph.org.cn (W.M.); gztt_2002@163.com (T.L.); jpengx@163.com (J.X.); letitiazeng@foxmail.com (W.Z.); lixing.echo@foxmail.com (X.L.); 3 Department of Epidemiology, College for Public Health & Social Justice, Saint Louis University, Saint Louis, MO 63104, USA; zqian2@slu.edu

**Keywords:** hypertension, domestic incense burning, indoor air pollution, China

## Abstract

Domestic incense burning is a common activity in China. Although it generates serious air pollution and has been linked to various health outcomes, it remains unknown whether it is associated with blood pressure and hypertension. A community-based survey including 1153 hypertensive subjects and 4432 normotensive participants in Guangdong (China) was used to examine this question. Two-level logistic regression was used to estimate the odds ratio (OR) and 95% confidence interval (CI). The analyses showed that, compared with non-users, OR of hypertension was 1.24 (95% CI: 1.03–1.50) for users, and 1.37 (95% CI: 1.04–1.80) for daily users with a clear dose-response relationship. The estimated increases in systolic and diastolic blood pressures were 1.02 mmHg (95% CI: 0.06–1.99) and 1.26 mmHg (95% CI: 0.69–1.83) for users, 0.67 mmHg (95% CI: −0.35–1.68) and 1.25 mmHg (95% CI: 0.66–1.85) for occasional users, and 2.09 mmHg (95% CI: 0.79–3.39) and 1.28 mmHg (95% CI: 0.52–2.05) for daily users, respectively. The results remained after adjusting for potential confounders and more pronounced associations were found among females. This study suggests that domestic incense burning may increase the risk of hypertension and blood pressure in the study population, and women are more vulnerable to these effects than men.

## 1. Introduction

Hypertension is a major public health problem with about one quarter of the population being affected in the world [1]. As one major risk factor for cardiovascular diseases, hypertension has been recognized as the most important cause of disability and the leading cause of death in the world [2].

Hypertension is one important public health issue not only for high income countries but also for developing countries, such as China, In China, the incidence of hypertension is expected to increase as a consequence of aging population and economic development [3,4]. It has been suggested that this increasing pattern cannot be fully attributed to genetic factors and changes in lifestyle and dietary factors, environmental factors, including air pollution, may also play an important role [5,6,7].

Growing evidence has suggested that indoor biomass burning is associated with elevated blood pressure [6,8,9] and risk of hypertension [5,6,10]. However, domestic use of coal and other solid fuels has been decreasing in many countries along with the socio-economic development [11]. As another important source of indoor air pollution, incense burning at home for ritual or religious purpose is an age-old custom among Chinese population [12]. Incense burning is a powerful source of a multitude of harmful constituents, including particulate matter and volatile organic compounds, which are potential air pollutants deleterious to human health [12,13,14,15,16]. Some studies have found that incense smoke has even higher toxic health effects than tobacco smoke [17], and has been linked with higher risk of cardiovascular mortality, respiratory tract carcinomas and nasopharyngeal cancer [18,19,20].

It is reasonable to hypothesize that exposure to indoor air pollution resulting from domestic incense burning is associated with higher blood pressure and risk of hypertension. In the present study we assessed the relationship of personal exposure to incense burning with blood pressure and hypertension in residents of Guangdong Province in southern China.

## 2. Materials and Methods

**Figure 1:**
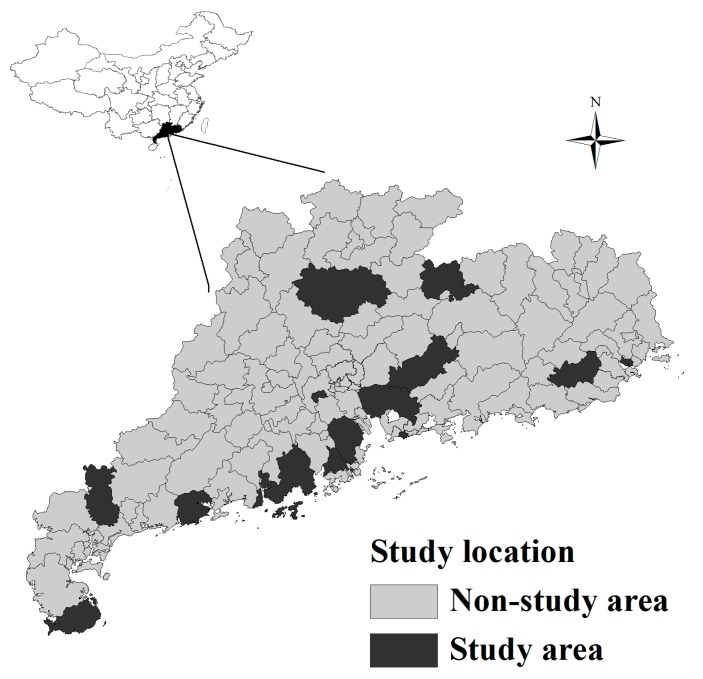
Geographical location of the study area in China.

We used information from a cross-sectional survey, which was conducted in 14 counties/districts of Guangdong Province, China (Figure 1). The survey was part of the China Chronic Disease and Risk Factor Surveillance, which was a series of surveys designed to investigate chronic diseases and related risk factors among residents over 18 years of age and has been shown to be representative of the whole population [21]. Details of the study design and data quality control have been described elsewhere [21,22]. Briefly, at the first stage, 14 counties/districts were randomly selected; these 14 counties/districts, spreading across all cities of the province, covered about 21% of the population and 15% of the province’s geographic area; for the second stage, two townships (in rural areas) or streets (urban areas) were randomly selected from each surveillance site; two administrative villages/communities were then randomly selected from each sampled township/street. In every selected village/community, at least 100 households were selected by simple random sampling method, and one subject was determined by the Kish grid method from each selected household. Only people who had lived in their current residence for six months or longer in the past 12 months were eligible to participate. Standard sampling protocols were used in all of the surveys.

The survey was conducted during the period from August to December 2013. Written informed consent was obtained before each interview. All eligible subjects were invited to attend face-to-face interviews and physical examinations. We used a standardized structured questionnaire to collect information mainly on basic socio-demographic characteristics, living condition, ventilation, domestic use of incense burning, and other potential confounding variables.

The arterial blood pressure was strictly measured in accordance with the 1999 World Health Organization/International Society of Hypertension Guidelines for the Management of Hypertension [23]. The standardized mercuric-column sphygmomanometers were used for the blood pressure measurement by trained investigators. Two consecutive blood pressure readings on right arms were taken after the participants rested in a seating position for at least 5 min, and the time interval between two measurements for each participant was about 2 min. The average of the two readings was used for the subsequent analyses.

In accordance with previous studies [4,5], hypertension was defined as an average systolic blood pressure (SBP) ≥140 mmHg or an average diastolic blood pressure (DBP) ≥90 mmHg, and participants who had a prior clinical diagnosis of hypertension before the interview were also classified as hypertension subjects.

Information on incense burning habits at each residence was collected in terms of number of days per month with incense burning at home. One day with incense burning was defined as burning at least one stick at home in a particular day. Two questions were used to assess the incense usage at home: (1) does your family burn incense at home? (2) if yes, how many days in one month do your family burn incense at home? Based on the distribution and the local custom, the frequency of domestic incense burning was classified into three categories in this study: daily usage, occasional usage and never. Daily usage was defined as burning incense at home on a daily basis (usually more than 25 days a month for the local people); occasional usage was defined as only burning incense at home for some special days, such as Spring Festival Day, Mid-autumn Day, often less than five days a month. Twenty-five days per month used as a cut-point was based on our field survey experience and the local custom, we also did sensitivity analyses by changing the cut-point to check the robustness of the results.

A number of covariate variables were collected, such as age, sex, weight, height, education, physical exercise, habits of tobacco smoking and alcohol consumption, dietary salt restriction, indoor biomass use, environmental tobacco smoking (ETS) exposure, and ventilation. Weight and height were measured when wearing light clothes but no shoes; body mass index (BMI) was calculated as weight in kilograms divided by height in meters squared. Ventilation was defined as whether they had ventilation facility used at their home. Physical exercise was defined as whether the subjects had a vigorous physical activity for at least 10 min each day. Dietary salt restriction was defined as whether they were undertaking salt intake restriction to limit daily salt intake less than 6 gram per individual as recommended by Chinese dietary guidelines [24]. To obtain the information of other indoor air pollution sources, the subjects were asked about their fuel type for domestic cooking, two fuel types were mainly used in the study population: clean fuels, including electricity and natural gas), and unclean fuels (such as coal, wood, dung and crop residues).

We also obtained the daily mean temperature data for the study counties/districts during the survey date from the publicly accessible China National Weather Data Sharing System (http://cdc.cma.gov.cn/home.do), as previous studies have suggested that ambient temperature was inversely associated with blood pressure [25].

Chi-square tests or Student’s t tests were used to examine the differences of socio-demographic factors between the hypertensive and normotensive participants. To investigate the relationship between hypertension and domestic incense burning, we used two-level logistic regression models which allowed for determination of the independent effects of incense burning on risk of hypertension, accounting for both individual- and area-level potential confounding factors [26]. In the model, the variables measured at the individual level were taken as the first-level units and those at the county/district level as the second-level units. At the individual level, we modeled the logit of the occurrence of hypertension as a function of incense burning after controlling for various individual-specific potential confounding factors. At the county/district level, we regressed the district-specific factors to explain the variations of hypertension occurrence, we used the annual per capita gross domestic product (GDP) as an indicator of the district-specific economic status in this study, as previous studies have reported a significant association between GDP and the risk of hypertension [27]. We selected the covariates in the final model based on two criteria: (1) they are known or suspected risk factors for hypertension or blood pressure according to the literature and our analyses; (2) these covariates, individually or in combination, lead to changes in the effect estimates by more than 10% for the predictor variables [28,29]. The final models were adjusted for a few covariates, including age, sex, BMI, education level, ETS exposure, cooking fuel type, ventilation, day of the week, and physical exercise. To consider the potential co-linearity caused by inclusion of two highly correlated variable in the same model, we checked the correlation between the included variables, if the correlation was higher than 0.80, they were not included in the same model [30]. In this study, the correlations between the variable included in the final model were lower than 0.80, so we included all the variable in the single model. The other variables (e.g., tobacco smoking, alcohol, or dietary salt) resulted in no further changes of point estimates and were not included in the models presented. For example, the estimated effects of increase burning were changed from odds ratio (OR) = 1.181 to OR = 1.182 when salt restriction was further included. Stratified analyses were conducted to investigate the possible effect modification by sex (male and female) and season. Season was categorized into warm and cold seasons, warm season was defined as May to October, and cold season was November to April.

We also examined the association of incense burning with systolic and diastolic blood pressures using generalized linear models, in the main model, we included all the participants; we also did a sensitivity analysis by excluding the subjects with prior diagnosis of hypertension, as their blood pressures might have been under control and cannot reflect the effects of exposure to incense burning.

One concern of our dataset was that we did not collect the information of family history of hypertension of the subjects, which might be one potential confounding factor for the association between indoor air pollution and hypertension [31]. To account for this uncertainty, we created a new variable to randomly simulate the status of family history of hypertension, which was based on one previous survey in Guangdong residents, which showed that the percentage of family history of hypertension among the hypertensive subjects and non-hypertensive subjects were about 25% and 19%, respectively (unpublished data). And then we did sensitivity analyses by including this variable in the models.

Approval to conduct this study was granted by the Medical Ethics Committee of National Centre for the Chronic and Noncommunicable Disease Control and Prevention, Chinese Centre for Disease Control and Prevention(ethics code: 201307). The methods were carried out in accordance with the approved guidelines. All the data management and statistical analyses were performed using R software Version 3.1.0 (R Development Core Team, Vienna, Austria). All statistical tests were two-sided and *p*-value < 0.05 was considered statistically significant.

## 3. Results

A total of 5600individuals were originally invited to participate in the cross-sectional survey, among which 5585 completed the interview, and were included in the subsequent analyses, yielding a response rate of 99.7%. Among them, 1153 were identified as hypertensive subjects based on the criteria described before. The distribution of socio-demographic characteristics and major risk factors of hypertension among hypertensive and normotensive subjects is shown in Table 1. Hypertensive subjects were statistically older than normotensive subjects (61.03 vs. 46.45 years, *p* = 0.01 for *t*-test); hypertensive subjects had higher BMI compared to normotensive subjects (23.59 kg/m^2^ vs. 22.36 kg/m^2^, *p* = 0.01 for *t*-test); more hypertensive subjects were males, had low education level, used unclean fuels for domestic cooking; fewer hypertensive participants were alcohol drinkers, had ever experienced environmental tobacco exposure, vigorous physical exercises, better ventilation at home. However the distribution of household income, ambient temperature on the survey date, smoking status, and dietary salt restriction was not statistically different among the two groups.

Table 2 shows the associations between domestic incense burning and the risk of hypertension in the univariate and multivariate two-level logistic regression models. More hypertensive subjects had the habit of incense burning at home than normotensive subjects, contributing to a crude OR of 1.24 (95% CI: 1.03–1.50). However, this association became non-significant after adjusting for potential confounding factors (the adjusted OR= 1.18 (95% CI: 0.97–1.44)). A significant association between the frequency of incense burning and hypertension risk was observed with a clear exposure-response relationship. The risk of hypertension who had daily practice of incense burning at home daily was 1.37 times of that in non-users (the crude OR= 1.37, 95% CI: 1.04–1.80), after controlling for the potential confounding factors, the association became non-significant with an OR being 1.27 (95% CI: 0.94–1.71), however the trend remained statistically significant. The sex-specific analyses showed that the effects remained only for females, for example, the ORs of domestic incense use were 1.18 (95% CI: 0.96–1.46) for males, and 1.50 (95% CI: 1.01–2.22) for females, respectively; and the ORs for the daily users were 1.29 (95% CI: 0.95–1.77) for males, and 1.81 (95% CI: 1.06–3.09) for females, respectively. The season-specific analyses (Table S1) showed positive associations in both seasons with a relative larger effect in cold season, however, the associations became statistically non-significant in the multivariate models. For the frequency of incense burning, we also observed approximately trend of larger effects among those with more usage of incense burning.

We also found significant associations of domestic incense burning with increased arterial blood pressures (Table 3).

Compared with non-users of incense, those with domestic incense burning had higher diastolic and systolic blood pressures, the estimates were 1.26 mmHg (95% CI: 0.69–1.83) and 1.02 mmHg (95% CI: 0.06–1.99), respectively. We also found a clear dose-response relationship between the frequency of domestic incense burning and the blood pressures, for example, people with daily exposure to incense burning had higher blood pressure, after controlling for potential confounding factors, the diastolic and systolic blood pressures among subjects with daily incense burning increased by 1.28 mmHg (95% CI: 0.5–2.05) and 2.09mmHg (95% CI: 0.79–3.39), respectively. Generally similar effects were observed for males and females, and generally more significant effects were observed for diastolic blood pressure; for instance, the estimated diastolic blood pressure effects of incense users were 0.95 (95% CI: 0.26–1.63) for males, 2.05 (95% CI: 1.00–3.10) for females, respectively; we did not find significant effect of incense burning exposure on systolic blood pressure among females, which might be due to relatively smaller sample size in this subgroup. In the sensitivity analyses, we found comparable effect estimates when controlling for the simulated family history of hypertension in the models (results not shown). Our analysis restricting to nonsmokers also yielded a similar result (Table S1).

## 4. Discussion

This was the first study to provide epidemiologic evidence of the effects of domestic incense burning on risk of hypertension and arterial blood pressure. The findings supported our hypothesis of the presence of an association of domestic incense burning with elevated risk of hypertension and arterial blood pressure, and it seemed that women were more vulnerable to these effects than men.

A few previous studies have linked exposure to indoor air pollution with risk of hypertension [5,6,9,10], while there is little information on the relationship between incense burning at home and measured blood pressure or hypertension risk, though it is a common practice among Chinese population. In this study, we found about 76.1% of the participants reported to be users of incense at home, which was comparable to the Chinese population living in Singapore [20] and higher than those in Hong Kong [19].

Findings from this study have some important public health implications. Though it is impractical to discourage this religious and ritual activity, findings from this study suggested that it is important to disseminate the results of the adverse health effects of incense burning to the users and provide appropriate methods to reduce the exposure and associated adverse health effects, and future studies are warranted to develop the least harmful types of incense as well as strategies to improve indoor air quality when burning incense indoors [16].

Though we observed that the hypertensive subjects were relatively older than the normotensive subjects, our further analysis found a comparable prevalence rate of incense burning among the older (over 50 years) and younger (<50 years) groups (79.1% in older group vs. 73.4% in younger group for incense burning) and (17.5% in older group vs.19.6% in younger group for daily incense burning), indicating that age should not have distorted the results to a great degree.

This study suggested a more pronounced hypertension associated with domestic incense burning among women. Similar findings have also been reported in previous studies on the effects of incense burning on lung carcinomas [18,32], and nasopharyngeal cancer [19]. Such findings could be explained by the fact that Chinese women usually spent more time at home, engaged in more ritual or religious activities than men and were more likely to be exposed to incense smoke at home [19]. A relatively higher effect was observed in cold season, one possible reason might be that during the cold season, the residents usually close their door and windows, leading to a higher indoor air pollution and personal exposure from incense burning. The underlying mechanism for the effects of exposure to incense burning on blood pressure and hypertension has not been well understood. Previous studies have suggested that the observed association may be attributable to long-term oxidative stress and systemic inflammation responses due to exposure to smoke from domestic incense burning. Inhalation of the air pollutants has been shown to result in oxidative stress [31,33], as well as systemic inflammation [34], which have been one important pathway of the development of hypertension [35]. The hypertension effects of exposure to incense burning could also be the result of a resetting of blood pressure-regulating mechanisms to higher levels as a result of repeated acute increases in blood pressure following inhalation of air pollutants from the incense burning [6]. autonomic tone change following air pollution exposure has been suggested to be the most likely pathway for the short-term increases in blood pressure increase [36]. The air pollution exposure could also induce the withdrawal of vagal activity and stimulation of the sympathetic nervous system, which could lead to systemic vasoconstriction, increased cardiac output, and increase in blood pressure [37]. Human experimental studies have also observed that exposure to air pollution could raise systolic and diastolic blood pressures [38,39]. In an experimental study conducted on pigs, Weber et al., found that carbon monoxide and other gas phase components of incense smoke might be responsible for the adverse health effects by inducing endothelial dysfunction [40]. Our finding was also supported by a randomized intervention study in Guatemalan women which found that transitioning from an open fire to an improved biomass stove was associated with lower blood pressure [41].

Some studies analyzing the emissions from incense burning have suggested volatile organic compounds and particulate matter to be the deleterious components [42,43,44,45]. This could raise the indoor concentrations of particulate matter less than 2.5 microns in aerodynamic diameter (PM_2.5_) to far exceed the ambient concentration guidelines proposed by the World Health Organization [46,47], and the volatile compounds included polycyclic aromatic hydrocarbons, oxygenated monoterpenes, esters, and formaldehydes [43,48,49], which have been reported to be associated with blood pressure and risk of hypertension. For example, one study examined the association between indoor PM_2.5_ exposure and blood pressure among women in rural China, and found a significant association between PM_2.5_ exposure and increased systolic and diastolic blood pressures [6]. In a cohort study from Germany, Fuks et al. [50] investigated the association of exposure to residential PM_2.5_ and PM_10_ with arterial blood pressure and hypertension, and found that per 3.9 μg/m^3^ increase in PM_10_ was associated with increase of 1.1 mmHg and 0.8 mmHg in systolic and diastolic blood pressures, respectively. Using a large database from three northeastern Chinese cities, Dong et al., investigated the association between various air pollutants and blood pressure and hypertension, and found that exposure to PM_10_ was significantly associated with increased blood pressure and higher risk of hypertension [5]. On the other hand, some other studies did not find any significant effects of air pollution on hypertension, such as studies from Taiwan [50,51] and Denmark [52].

The main strength of this study included its population-based study design, relatively large sample size, detailed information on lifestyle factors that were established or potential risk factors of hypertension. The use of domestic incense was quite common in the participants, providing a good opportunity to evaluate the association between incense use and hypertension, which has not been reported previously.

A few limitations should be noted. First, due to cross-sectional nature of the study design, residual or unmeasured confounding was possible, and causality should be inferred with caution. Because incense burning was one practice related to religious and cultural brief, it was possible that there were some differences in other characteristics between the participants who used incense and those who did not, resulting in potential unmeasured confounding factors in our analysis. Second, selection and information biases were possible. However, we have attempted to reduce these biases as much as possible through strict quality control and assurance processes. The interviewers were strictly trained to conduct survey with the same degree of questioning for all participants, and the study was introduced as a general health study. Third, the information of family history of hypertension was not available to our analysis; our sensitivity analysis based on simulated information suggested that it should not have affected our result to a great extent. Finally, we did not collect the information on some important factors that were likely to influence the results, such as the type of incense, the duration of incense burning, isolation between incense burning room and living room, exposures at temples, time spent at home each day, survey time (morning or evening), noise, anxiety, psychological stress, work stress, anti-hypertensive medications and fasting status, more studies are needed to further investigate this research question.

## 5. Conclusions

In summary, our study provides epidemiologic evidence that exposure to smoke from domestic incense burning may contribute to the risk of hypertension and increased blood pressure, and women were more vulnerable to these effects than man.

## Figures and Tables

**Table 1:** Comparison of socio-demographic and major risk factors between 1153 hypertensive subjects and 4432 normotensive subjects in Guangdong, China.

Variables	Hypertensive (*N* (%))	Normotensive (*N* (%))	*p*-Value *
Age (years, mean (SD))	61.03 (13.87)	46.45 (15.27)	0.01
Sex			
Male	817 (70.86)	2838 (64.04)	
Female	336 (29.14)	1594 (35.97)	0.01
BMI (kg/m^2^)	23.59 (3.44)	22.36 (2.84)	0.01
Monthly household income (RMB)	4670 (7814)	4947 (5771)	0.31
Ambient temperature (°C)	22.40 (5.37)	22.48 (5.01)	0.63
Education			
Low	732 (63.49)	1717 (38.74)	
High	421 (36.51)	2715 (61.26)	0.01
Smoking status			
Nonsmoker	856 (74.24)	3279 (73.98)	
Smoker	297 (25.76)	1153 (26.02)	0.89
ETS exposure **^#^**			
Never	455 (39.46)	1584 (35.74)	
Ever	698 (60.54)	2848 (64.26)	0.02
Drinking status			
Nondrinking	852 (73.89)	3140 (70.85)	
Drinking	301 (26.11)	1292 (29.15)	0.05
Physical exercise			
No	879 (76.24)	2847 (64.25)	
Yes	274 (23.76)	1584 (35.75)	0.01
Indoor fuel type			
Clean	879 (76.24)	3601 (81.25)	
Unclean	274 (23.76)	831 (18.75)	0.01
Ventilation			
No	197 (17.09)	619 (13.97)	
Yes	956 (82.91)	3813 (86.03)	0.01
Salt restriction			
No	1070 (92.80)	4133 (93.25)	
Yes	82 (7.20)	299 (6.75)	0.70

****^#^**** ETS: environmental tobacco smoke; * χ2 tests for categorical variables and *t*-tests for continuous variables.

**Table 2:** Association between domestic incense burning and hypertension risk.

Variables	Hypertensive (*N* = 1153)	Normotensive (*N* = 4432)	Crude OR (95% CI)	*p*-Value	Adjusted OR* (95% CI)	*p*-Value
Overall						
Incense Burning						
No	250 (21.68)	1086 (24.50)	Reference		Reference	
Yes	903 (78.32)	3346 (75.50)	1.24 (1.03–1.50)	0.02	1.18 (0.97–1.44)	0.10
Frequency of Incense Burning						
Never	250 (21.68)	1086 (24.50)	Reference		Reference	
Occasional	663 (57.50)	2634 (59.43)	1.21 (0.99–1.48)	0.06	1.16 (0.94–1.43)	0.18
Daily	240 (20.82)	712 (16.06)	1.37 (1.04–1.80)	0.03	1.27 (0.94–1.71)	0.11
P for trend			0.02		0.05	
Male						
Incense Burning						
No	206 (25.21)	810 (28.54)	Reference		Reference	
Yes	611 (74.79)	2028 (71.46)	1.18 (0.96–1.46)	0.12	1.11 (0.88–1.39)	0.39
Frequency of Incense Burning						
Never	206 (25.21)	810 (28.54)	Reference		Reference	
Occasional	429 (52.51)	1508 (53.14)	1.15 (0.95–1.77)	0.24	1.08 (0.85–1.37)	0.55
Daily	182 (22.28)	520 (18.32)	1.29 (0.95–1.77)	0.11	1.20 (0.86–1.68)	0.29
P for trend			0.09		0.29	
Female						
Incense Burning						
No	44 (13.10)	276 (17.31)	Reference		Reference	
Yes	292 (86.90)	1318 (82.69)	1.50 (1.01–2.22)	0.04	1.45 (0.96–2.20)	0.08
Frequency of Incense Burning						
Never	44 (13.10)	276 (17.31)	Reference		Reference	
Occasional	234 (69.64)	1126 (70.64)	1.43 (0.96–2.14)	0.08	1.39 (0.91–2.12)	0.12
Daily	58 (17.26)	192 (12.05)	1.81 (1.06–3.09)	0.03	1.79 (1.01–3.16)	0.04
P for trend			0.03		0.04	

* Adjusted for age, sex, BMI, ETS exposure, cooking fuel type, ventilation, education level, day of the week, and physical exercise, and county/district-specific GDP in the two-level logistic regression models.

**Table 3:** Estimated absolute increase in mean arterial blood pressures (mmHg) with 95% CI associated with domestic incense burning.

Variable	Diastolic Blood Pressure		Systolic Blood Pressure	
	Estimate * (95% CI)	*p*-Value	Estimate * (95% CI)	*p*-Value
Overall				
Incense burning				
No	Reference		Reference	
Yes	1.26 (0.69–1.83)	0.01	1.02 (0.06–1.99)	0.04
Frequency of incense burning				
Never	Reference		Reference	
Occasional	1.25 (0.66–1.85)	0.01	0.67 (−0.35–1.68)	0.20
Daily	1.28 (0.52–2.05)	0.01	2.09 (0.79–3.39)	0.01
P for trend	0.01		0.01	
Males				
Incense burning				
No	Reference		Reference	
Yes	0.95 (0.26–1.63)	0.01	0.89 (−0.27–2.05)	0.13
Frequency of incense burning				
Never	Reference		Reference	
Occasional	0.81 (0.08–1.53)	0.03	0.30 (−0.93–1.52)	0.63
Daily	1.30 (0.40–2.21)	0.01	2.36 (0.83–3.89)	0.01
P for trend	0.01		0.01	
Females				
Incense burning				
No	Reference		Reference	
Yes	2.05 (1.00–3.10)	0.01	1.23 (−0.55–3.02)	0.17
Frequency of incense burning			
Never	Reference		Reference	
Occasional	2.20 (1.13–3.26)	0.01	1.15 (−0.66–2.97)	0.21
Daily	1.28 (−0.19–2.74)	0.09	1.66 (−0.82–4.14)	0.19
P for trend	0.03		0.17	

* Adjusted for age, sex, BMI, cooking fuel type, ventilation, education level, day of the week, and physical exercise in the multivariate regression models.

## Supplementary Materials

The following are available online at www.mdpi.com/1660-4601/14/7/788/s1, Table S1: Season-specific association between domestic incense burning and hypertension risk.
