# Supplementary material for: The Association of Domestic Incense Burning with Hypertension and Blood Pressure in Guangdong, China
Source: Int J Environ Res Public Health. 2017 Jul 14;14(7):788. doi: 10.3390/ijerph14070788 (PMC5551226; doi:10.3390/ijerph14070788)
Supplement: Supplementary file 1 [file ijerph-14-00788-s001.pdf]

Table s1. Season-specific association between domestic incense burning and hypertension risk

Variable	Crude OR (95% CI)	*P* value	Adjusted OR * (95% CI)	P value
**Warm (May-October)**				
**Incense burning**				
No	Reference		Reference	
Yes	1.15 (0.91 -1.47)	0.24	1.11 (0.86-1.44)	0.40
**Frequency of incense burning**				
Never	Reference		Reference	
Occasional	1.08 (0.84-1.39)	0.56	1.06 (0.81-1.38)	0.66
Daily	1.43 (1.01-2.02)	0.04	1.31 (0.91-1.91)	0.15
P for trend	0.06		0.19	
**Cold (November-April)**				
**Incense burning**				
No	Reference		Reference	
Yes	1.37 (1.02-1.84)	0.04	1.26 (0.92-1.72)	0.15
**Frequency of incense burning**				
Never	Reference		Reference	
Occasional	1.39 (1.02-1.89)	0.04	1.25 (0.90-1.73)	0.19
Daily	1.31 (0.86-2.01)	0.21	1.30 (0.82-2.05)	0.26
P for trend	0.09		0.18	

*Adjusted for age, sex, BMI, ETS exposure, cooking fuel type, ventilation, education level, and physical exercise, and county/district-specific GDP in the two-level logistic regression models.
